# Supplementary material for: Attitudes of healthcare professionals in treatment decision-making for older adults with cancer: a scoping review protocol
Source: BMJ Open. 2023 Oct 11;13(10):e077628. doi: 10.1136/bmjopen-2023-077628 (PMC10582975; doi:10.1136/bmjopen-2023-077628)
Supplement: Author's manuscript [file bmjopen-2023-077628.draft_revisions.pdf]

BMJ Open is committed to open peer review. As part of this commitment we make the peer review history of every article we publish publicly available.

When an article is published we post the peer reviewers' comments and the authors' responses online. We also post the versions of the paper that were used during peer review. These are the versions that the peer review comments apply to.

The versions of the paper that follow are the versions that were submitted during the peer review process. They are not the versions of record or the final published versions. They should not be cited or distributed as the published version of this manuscript.

BMJ Open is an open access journal and the full, final, typeset and author-corrected version of record of the manuscript is available on our site with no access controls, subscription charges or pay-per-view fees (http://bmjopen.bmj.com).

If you have any questions on BMJ Open's open peer review process please email info.bmjopen@bmj.com

BMJ Open

The attitudes of Healthcare Professionals in treatment decision making for older adults with cancer: A scoping review protocol

Journal:	*BMJ Open*
Manuscript ID	bmjopen-2023-077628
Article Type:	Protocol
Date Submitted by the Author:	10-Jul-2023
Complete List of Authors:	Pinker, India; Luxembourg Institute of Health, Department of Precision Health<br>Pilleron, Sophie; Luxembourg Institute of Health, Department of Precision Health
Keywords:	Aged, ONCOLOGY, Clinical Decision-Making, Protocols & guidelines < HEALTH SERVICES ADMINISTRATION & MANAGEMENT, Physicians

SCHOLARONE™
Manuscripts

The attitudes of Healthcare Professionals in treatment decision making for older adults with cancer: A scoping review protocol

Authors

India Pinker[1],Sophie Pilleron[1]

[1] Ageing, Cancer, and Disparities Research Unit, Department of Precision Health, Luxembourg Institute of Health, 1A-B, rue Thomas Edison, 1445 Strassen, Luxembourg

Author's contact: india.pinker@lih.lu

For peer review only

Abstract

Introduction:

The global population is ageing, and the number of older adults with cancer is increasing worldwide. These patients have unique care needs, and healthcare professionals (HCPs) often have to rely on their own attitudes and assumptions to make a decision due to limited evidence. Differences in patient and HCP attitudes can impact treatment decisions and patient outcomes. There is limited research, however, on HCP attitudes in treatment decision making for older adults with cancer. This scoping review aims to explore the attitudes of HCPs in treatment decision making for older adults with cancer.

Methods and Analysis:

The electronic databases PubMed, Elsevier Embase, Medline (from Embase) and EBSCO CINAHL Complete will be searched on the 4th of July 2023 to identify eligible studies based upon the developed inclusion and exclusion criteria. No restrictions on study period, geography or language will be applied. Screening and data extraction will be completed independently by teams of two reviewers, with conflicts resolved by a 3rd reviewer. The review findings will be presented as tables and in a narrative summary.

This scoping review follows the framework of Arksey and O'Malley with the Levac extension. Data extraction and analysis will be performed to identify patterns and gaps in the literature to provide an overview of the attitudes of HCPs in treatment decision making for older adults with cancer.

Ethics and Dissemination:

No ethical approval is needed. The findings will be published in a peer-reviewed journal and presented at conferences, providing insights to improve treatment decision making for older adults with cancer and guide future interventions for HCPs in geriatric oncology.

Registration Details: Registered on Open Science Framework at https://doi.org/10.17605/OSF.IO/T7FD3

Strengths and Limitations

- The search strategy for this scoping review was devised by a research librarian, enhancing the robustness of the search.
- The search was not restricted in terms of language, geography, or time, resulting in a more comprehensive search.
- A limitation of the study, however, is the difficulty in measuring and defining the primary target outcome of attitude, potentially leading to variations in interpretation.
- As it is a scoping review there is no assessment of the quality of the papers included.

For peer review only

Introduction:

Reflecting population growth, the number of older adults within the global population is steadily rising. This demographic trend is also reflected in cancer incidence with half of these cases diagnosed in patients over the age of 65 (1). Older adults with cancer can have unique care management needs because of comorbidity, polypharmacy and frailty (2, 3). Further complicating matters, older adults are a notably heterogeneous group with regard to health and fitness and are underrepresented in clinical trials, limiting the evidence base from which Healthcare Professionals (HCPs) can draw treatment decisions (4). Consequently, HCPs may rely on their attitudes, preferences and assumptions. Attitudes are ascribed a functional role in guiding behaviour, particularly when confronted with decision-making under conditions of uncertainty (5). Reaching agreement on treatment decisions under these conditions may, therefore, be more complex when attitudes differ between HCPs and patients (6).

Attitudes are reflected in an individual's preferences, inclinations and tendencies (5). When patient and HCP attitudes and consequent goals differ, this can impact patient representation in treatment decision making (7). The significance of this issue may be heightened in cancer cases, where decisions frequently involve multidisciplinary teams (MDTs) without direct patient involvement. For these cases, HCPs serve as proxies for the patient, emphasising the importance of aligning the understanding of both HCP and patient preferences. If there are discrepancies in priorities between the two, health management decisions may fail to adequately reflect the holistic needs of the patient (8). This, in turn, could ultimately impact the cancer outcomes of the patient as they may not be as invested or adhere to treatment that does not represent their needs or preferences (9).

HCPs often lack sufficient training that fosters a deliberate exploration of older adults preferences during the decision-making phase of treatment. This may lead to the tendency of assuming patient values based on the HCP's personal attitude – informed by their preferences, experiences, and values - which may not accurately reflect patient perspectives (5, 11).

While most studies and recent reviews have focused on older adults' preferences or patient-HCP comparisons (6, 8-13), there is limited research on oncology-based HCP preferences alone.

As our understanding of the older adults with cancer's preferences develops, it is important to clarify the HCP perspective to ensure that the treatment decision process – which is often different for older adults compared to younger adults (12) – is not inhibited by incompatible goals. Consequently, gaining an understanding of the patterns within these attitudes is crucial to identify strategies that can effectively engage healthcare professionals and ultimately benefit older adults with cancer.

For peer review only

Objectives:

This scoping review aims to provide an overview of the attitudes of Healthcare Professionals in treatment decision making related to older adults with cancer.

Specifically:

- Identify patterns in attitudes of HCPs specific to treatment decision making for older adults with cancer
- Identify potential contributing factors to these attitudes
- Highlight gaps in the literature

Methods and analysis:

This protocol is written based on PRISMA ScR guidelines for Scoping reviews (14).

Design

A scoping review will be conducted based on the Arksey and O'Malley methodological framework with the extension developed by Levac and colleagues (15, 16). The reporting of the review will be based on PRISMA ScR guidelines for Scoping reviews (14). A preliminary search - conducted in May 2023 - of PubMed, the Cochrane Database of Systematic Reviews, PROSPERO and Open Science Framework was conducted and no current or underway systematic reviews or scoping reviews on the topic were identified.

Protocol Registration

The final protocol is registered on the Open Science Framework at https://doi.org/10.17605/OSF.IO/T7FD3

Eligibility Criteria

The eligibility criteria was formulated based on the PICO Framework (17), see Table 1.

For peer review only

Table 1: Eligibility criteria based on the PICO framework

Element	Inclusion Criteria	Exclusion Criteria
**P**articipants	Papers involving Healthcare Professionals (e.g. Oncologists, nurses, physicians, nurse practitioners…) of Older Adults (mean/median ≥65 years old) with cancer, any HCP affiliated with cancer care or treatment decisions in cancer care of older adults.	HCPs in the study are exclusively associated with patients younger than 65 years old (for example, paediatric, patient-HCP comparative studies where mean/median patient age <65).
**I**ntervention or exposure of interest	The primary focus of the studies must be on the HCP perspective of treatment decision making in the field of geriatric oncology. Furthermore, the outcomes of the studies should include information regarding subjective inclinations, rankings and perspectives that influence the choices made within the decision-making process. This will be indicative of HCP preferences, attitudes and their individual perspectives.	
**C**omparator	Different HCPs (e.g. Oncologists and specialist nurses). Or different age groups – but this must include a group where the adults are ≥65 years old.<br>Comparisons between patient and HCP priorities, only HCP priorities will be extracted from these studies. Comparisons are not mandatory for inclusion.	None

For peer review only

Outcome	Any outcomes related to individual professional factors within treatment decision making in geriatric oncology such as attitudes, perceptions, priorities, or preferences.<br><br>For the purposes of this review, 'attitudes' refers to any subjective inclinations, rankings, and perspectives that influence the choices made when determining the most appropriate course of treatment for their older patients. This description may be encompassed by terms such as perceptions, priorities, or preferences.	
Study Design	Any quantitative design<br>Any qualitative design<br>Mixed methods designs<br>All types of reviews (i.e. literature, narrative, systematic…) to synthesise commonly identified inclinations of HCPs during the treatment decision making process.	Case reports
Article Type	Original peer-reviewed research articles.	Perspective or opinion pieces, book chapters, dissertations, editorials, protocols, conference abstracts
Language	No restriction	
Study Period	No restriction	
Geography	No restriction	

126

For peer review only

Information Resources

We will search for papers from the following databases:

- PubMed
- Elsevier Embase
- Medline (from Embase)
- EBSCO CINAHL Complete

Search

The search strategy was developed by a health focused research librarian. Comprehensive search strategies will be employed in each of the databases, utilising a combination of interface command language and text key-words. The search will be formulated in PubMed, from which the strategy will be translated into other databases reflecting their command languages if this is applicable. The search strategy will be made using Boolean operators and truncation. All searches will be reported within PRISMA-ScR guidelines (16).

Table 2: Key concepts and preliminary search terms

**Key concept**	**Search Terms**	**MeSH Terms**
Healthcare professionals	"healthcare professional*" OR "health care professional*" OR "health* professional*" OR "health profession*"	Health Personnel
Treatment decision making	"decision making", "treatment decision", "decision making" AND treatment	Decision Making, Clinical Decision-Making
Older adults	Aged, "Older adult*", Elderly, Geriatric, Nonagenarian, Octogenarian, Centenarian	Aged
Cancer	Cancer*, neoplasm*, tumour*, tumor*, Neoplasia*, Malignanc*, *carcinom*, sarcom*	Neoplasms

For peer review only

Due to the limited number of hits from the above search, no search terms for the key concept of 'attitudes' were included.

Study Selection

The selection of studies will follow a multi-stage process:

- *Screening by Title and Abstract*: Prior to primary screening, duplicate records will be removed. Titles will be screened by independent review done in a reviewer pair. Disagreements regarding the inclusion of papers for full text review will be resolved by a third reviewer.
- *Screening by Full text:* Full texts of all papers included after the title and abstract screening will be acquired and assessed by the reviewer pair independently, judging whether the papers meet the inclusion criteria. Disagreements will be resolved through the same process as the title and abstract screening.
- *Screening by References:* The reference list of all included full papers will be screened by the reviewers.

Screening will be streamlined by using a specific review software, Rayyan (18).

Data Management (included and excluded papers)

All papers considered for full text screening during the abstract and title screening process that are then excluded during the full text screening will be tabulated on a digital spreadsheet with a justification for exclusion based upon the inclusion and exclusion criteria. Endnote20 will be used for reference management and bibliography generation.

Abstract-Only Cases

In cases where only the abstract of a potentially eligible paper is available, the authors of this paper will be contacted inquiring whether a full version of the manuscript has been published elsewhere. If it has not been published elsewhere, the study will be excluded. The time limit placed on this correspondence will be 2 weeks. Following this, one follow up email will be sent with the same correspondence time limit. If there is no response, the paper will be excluded.

Data collection process and extraction

Software will be used to compile the reviewer team's extracted data. For every included paper, two reviewers will independently extract the following data as a minimum:

- Author(s)
- Year of publication

For peer review only

- Study objective
- Study design (e.g. qualitative, mixed methods, longitudinal etc.)
- Country of study
- Sampling method (e.g. convenience, snowballing etc.)
- Sample size
- Sample description (sex/gender, age, health profession etc.)
- Description of attitudes if provided
- Descriptions of factors accounted for in the decision making process if provided
- Analytical approach used (e.g. statistical modelling, thematic analysis etc.)
- Summary of main findings

For disagreement over any data point, the same review process followed during screening will be employed.

All collected data will be tabulated for presentation in the written report, accompanied by a narrative summary relating the results to provide an overview of the attitudes of HCP in treatment decision making related to older adults with cancer.

<u>Assessment of Quality</u>

No quality assessment for included papers will be conducted, as suggested by both the Arksey and O'Malley Framework and Joanna Briggs Institute (14, 19).

Implications:

Findings of this review will provide insight into areas of focus to improve the treatment decision making process for older adults with cancer, including variables that may be valuable in the design of future interventions for HCPs involved in geriatric oncology.

Ethics and Dissemination:

No ethical approval is required for this research as it involves the analysis of secondary data. The results of this review will be published in a peer-reviewed journal and presented at conferences.

References:

1. Pilleron S, Sarfati D, Janssen-Heijnen M, Vignat J, Ferlay J, Bray F, et al. Global cancer incidence in older adults, 2012 and 2035: A population-based study. International Journal of Cancer. 2019;144(1):49-58.

2. Berben L, Floris G, Wildiers H, Hatse S. Cancer and Aging: Two Tightly Interconnected Biological Processes. Cancers. 2021;13(6):1400.

For peer review only

3. Boakye D, Rillmann B, Walter V, Jansen L, Hoffmeister M, Brenner H. Impact of comorbidity and frailty on prognosis in colorectal cancer patients: A systematic review and meta-analysis. Cancer Treatment Reviews. 2018;64:30-9.

4. Outlaw D, Williams GR. Is the lack of evidence in older adults with cancer compromising safety? Expert Opinion on Drug Safety. 2020;19(9):1059-61.

5. Riemer H, Shavitt S, Koo M, Markus HR. Preferences don't have to be personal: Expanding attitude theorizing with a cross-cultural perspective. Psychological Review. 2014;121:619-48.

6. Montgomery AA, Fahey T. How do patients' treatment preferences compare with those of clinicians? Qual Health Care. 2001;10 Suppl 1(Suppl 1):i39-43.

7. Lamb B, Mostafid H, Vincent C, Green J. Quality improvement in multidisciplinary cancer teams: an investigation of teamwork and clinical decision-making and cross-validation of assessments. Annals of surgical oncology. 2011;18:3535-43.

8. Sathanapally H, Sidhu M, Fahami R, Gillies C, Kadam U, Davies MJ, et al. Priorities of patients with multimorbidity and of clinicians regarding treatment and health outcomes: a systematic mixed studies review. BMJ Open. 2020;10(2):e033445.

9. Smaje A, Weston-Clark M, Raj R, Orlu M, Davis D, Rawle M. Factors associated with medication adherence in older patients: A systematic review. AGING MEDICINE. 2018;1(3):254-66.

10. Seghers P, Wiersma A, Festen S, Stegmann ME, Soubeyran P, Rostoft S, et al. Patient Preferences for Treatment Outcomes in Oncology with a Focus on the Older Patient-A Systematic Review. Cancers (Basel). 2022;14(5).

11. Rocque GB, Rasool A, Williams BR, Wallace AS, Niranjan SJ, Halilova KI, et al. What Is Important When Making Treatment Decisions in Metastatic Breast Cancer? A Qualitative Analysis of Decision-Making in Patients and Oncologists. The Oncologist. 2019;24(10):1313-21.

12. Step MM, Siminoff LA, Rose JH. Differences in Oncologist Communication Across Age Groups and Contributions to Adjuvant Decision Outcomes. Journal of the American Geriatrics Society. 2009;57(s2):s279-s82.

13. Sifer-Rivière L, Girre V, Gisselbrecht M, Saint-Jean O. Physicians' perceptions of cancer care for elderly patients: a qualitative sociological study based on a pilot geriatric oncology program. Crit Rev Oncol Hematol. 2010;75(1):58-69.

14. . Tricco AC, Lillie E, Zarin W, O'Brien KK, Colquhoun H, Levac D, et al. PRISMA Extension for Scoping Reviews (PRISMA-ScR): Checklist and Explanation. Ann Intern Med. 2018;169(7):467-73.

For peer review only

15. Arksey H, O'Malley L. Scoping studies: towards a methodological framework. International Journal of Social Research Methodology. 2005;8(1):19-32.

16. Levac D, Colquhoun H, O'Brien KK. Scoping studies: advancing the methodology. Implementation Science. 2010;5(1):69.

17. Schardt C, Adams MB, Owens T, Keitz S, Fontelo P. Utilization of the PICO framework to improve searching PubMed for clinical questions. BMC Med Inform Decis Mak. 2007;7:16.

18. Ouzzani M, Hammady H, Fedorowicz Z, Elmagarmid A. Rayyan—a web and mobile app for systematic reviews. Systematic reviews. 2016 Dec;5:1-0.

19. Peters MD, Godfrey CM, Khalil H, McInerney P, Parker D, Soares CB. Guidance for conducting systematic scoping reviews. Int J Evid Based Healthc. 2015;13(3):141-6.

Authors' contributions:

The scoping review protocol was written up by India Pinker and reviewed by Sophie Pilleron.

Funding

This work is supported by the Luxembourg National Research Fund (FNR), Project n°16731054. The funder did not play a role in the write up of this protocol.

Competing Interests

There are no competing interests to declare.

For peer review only

PRISMA-P 2015 Checklist

This checklist has been adapted for use with protocol submissions to *Systematic Reviews* from Table 3 in Moher D et al: Preferred reporting items for systematic review and meta-analysis protocols (PRISMA-P) 2015 statement. *Systematic Reviews* 2015 **4**:1

Section/topic	#	Checklist item	Information reported Yes	Information reported No	Line number(s)
**ADMINISTRATIVE INFORMATION**					
**Title**					
Identification	1a	Identify the report as a protocol of a systematic review	☒	☐	2
Update	1b	If the protocol is for an update of a previous systematic review, identify as such	☐	☒	Not appropriate
**Registration**	2	If registered, provide the name of the registry (e.g., PROSPERO) and registration number in the Abstract	☒	☐	49-50
**Authors**					
Contact	3a	Provide name, institutional affiliation, and e-mail address of all protocol authors; provide physical mailing address of corresponding author	☒	☐	5, 10-11, 15
Contributions	3b	Describe contributions of protocol authors and identify the guarantor of the review	☒	☐	255
**Amendments**	4	If the protocol represents an amendment of a previously completed or published protocol, identify as such and list changes; otherwise, state plan for documenting important protocol amendments	☐	☒	Not appropriate
**Support**					
Sources	5a	Indicate sources of financial or other support for the review	☒	☐	257
Sponsor	5b	Provide name for the review funder and/or sponsor	☒	☐	257
Role of sponsor/funder	5c	Describe roles of funder(s), sponsor(s), and/or institution(s), if any, in developing the protocol	☒	☐	258
**INTRODUCTION**					
**Rationale**	6	Describe the rationale for the review in the context of what is already known	☒	☐	61-94
**Objectives**	7	Provide an explicit statement of the question(s) the review will address with reference to participants, interventions, comparators, and outcomes (PICO)	☒	☐	97-103
**METHODS**					

BioMed Central The Open Access Publisher

2

Section/topic	#	Checklist item	Information reported Yes	Information reported No	Line number(s)
**Eligibility criteria**	8	Specify the study characteristics (e.g., PICO, study design, setting, time frame) and report characteristics (e.g., years considered, language, publication status) to be used as criteria for eligibility for the review	☒	☐	118 - 126
**Information sources**	9	Describe all intended information sources (e.g., electronic databases, contact with study authors, trial registers, or other grey literature sources) with planned dates of coverage	☒	☐	127 - 132
**Search strategy**	10	Present draft of search strategy to be used for at least one electronic database, including planned limits, such that it could be repeated	☒	☐	134 - 144
STUDY RECORDS					
Data management	11a	Describe the mechanism(s) that will be used to manage records and data throughout the review	☒	☐	158 -162
Selection process	11b	State the process that will be used for selecting studies (e.g., two independent reviewers) through each phase of the review (i.e., screening, eligibility, and inclusion in meta-analysis)	☒	☐	145 - 158
Data collection process	11c	Describe planned method of extracting data from reports (e.g., piloting forms, done independently, in duplicate), any processes for obtaining and confirming data from investigators	☒	☐	169 – 171 & 186 - 190
**Data items**	12	List and define all variables for which data will be sought (e.g., PICO items, funding sources), any pre-planned data assumptions and simplifications	☒	☐	172 - 185
**Outcomes and prioritization**	13	List and define all outcomes for which data will be sought, including prioritization of main and additional outcomes, with rationale	☒	☐	172 - 185
**Risk of bias in individual studies**	14	Describe anticipated methods for assessing risk of bias of individual studies, including whether this will be done at the outcome or study level, or both; state how this information will be used in data synthesis	☒	☐	191 - 193
DATA					
**Synthesis**	15a	Describe criteria under which study data will be quantitatively synthesized	☐	☒	Not appropriate
	15b	If data are appropriate for quantitative synthesis, describe planned summary measures, methods of handling data, and methods of combining data from studies, including any planned exploration of consistency (e.g., I^2, Kendall's tau)	☐	☒	Not appropriate
	15c	Describe any proposed additional analyses (e.g., sensitivity or subgroup analyses, meta-regression)	☐	☒	Not appropriate
	15d	If quantitative synthesis is not appropriate, describe the type of summary planned	☒	☐	188 -190
**Meta-bias(es)**	16	Specify any planned assessment of meta-bias(es) (e.g., publication bias across studies, selective reporting within studies)	☒	☐	191 - 193
**Confidence in cumulative evidence**	17	Describe how the strength of the body of evidence will be assessed (e.g., GRADE)	☒	☐	191 - 193

For peer review only

BioMed Central The Open Access Publisher

BMJ Open

The attitudes of Healthcare Professionals in treatment decision making for older adults with cancer: A scoping review protocol

Journal:	*BMJ Open*
Manuscript ID	bmjopen-2023-077628.R1
Article Type:	Protocol
Date Submitted by the Author:	18-Sep-2023
Complete List of Authors:	Pinker, India; Luxembourg Institute of Health, Department of Precision Health<br>Pilleron, Sophie; Luxembourg Institute of Health, Department of Precision Health
**Primary Subject Heading**:	Geriatric medicine
Secondary Subject Heading:	Communication, Oncology, Patient-centred medicine, Public health, Sociology
Keywords:	Aged, ONCOLOGY, Clinical Decision-Making, Protocols & guidelines < HEALTH SERVICES ADMINISTRATION & MANAGEMENT, Physicians

SCHOLARONE™
Manuscripts

The attitudes of Healthcare Professionals in treatment decision making for older adults with cancer: A scoping review protocol

Authors

India Pinker[1],Sophie Pilleron[1]

[1] Ageing, Cancer, and Disparities Research Unit, Department of Precision Health, Luxembourg Institute of Health, 1A-B, rue Thomas Edison, 1445 Strassen, Luxembourg

Author's contact: india.pinker@lih.lu

For peer review only

Abstract

Introduction:

The number of older adults with cancer is increasing worldwide. These patients' unique care needs, arising from comorbidity, polypharmacy, and frailty, often necessitate healthcare professionals (HCPs) to rely on their own attitudes and assumptions to a greater extent when making decisions due to limited evidence. Differences in patient and HCP attitudes can impact treatment decisions and patient outcomes. There is limited research, however, on HCP attitudes in treatment decision making for older adults with cancer. This scoping review aims to explore the attitudes of HCPs in treatment decision making for older adults with cancer.

Methods and Analysis:

The electronic databases PubMed, Elsevier Embase, Medline (from Embase) and EBSCO CINAHL Complete will be searched on the 4th of July 2023 to identify eligible studies based upon the developed inclusion and exclusion criteria. No restrictions on study period, geography or language will be applied. Screening and data extraction will be completed independently by teams of two reviewers, with conflicts resolved by a 3rd reviewer. The review findings will be presented as tables and in a narrative summary.

This scoping review follows the framework of Arksey and O'Malley with the Levac extension. Data extraction and analysis will be performed to identify patterns and gaps in the literature to provide an overview of the attitudes of HCPs in treatment decision making for older adults with cancer.

Ethics and Dissemination:

No ethical approval is needed. The findings will be published in a peer-reviewed journal and presented at conferences, providing insights to improve treatment decision making for older adults with cancer and guide future interventions for HCPs in geriatric oncology.

Registration Details: Registered on Open Science Framework at https://doi.org/10.17605/OSF.IO/T7FD3

Strengths and Limitations

- The search strategy for this scoping review was devised by a research librarian, enhancing the robustness of the search.
- The search was not restricted in terms of language, geography, or time, resulting in a more comprehensive search.
- A limitation of the study, however, is the difficulty in measuring and defining the primary target outcome of attitude, potentially leading to variations in interpretation.
- As it is a scoping review there is no assessment of the quality of the papers included.

For peer review only

Introduction:

Reflecting population growth, the number of older adults within the global population is steadily rising. This demographic trend is also reflected in cancer incidence with half of these cases diagnosed in patients over the age of 65 (1). Older adults with cancer can have unique care management needs because of comorbidity, polypharmacy and frailty (2, 3). Further complicating matters, older adults are a notably heterogeneous group with regard to health and fitness and are underrepresented in clinical trials, limiting the evidence base from which Healthcare Professionals (HCPs) can draw treatment decisions (4). Consequently, HCPs may rely on their attitudes, preferences and assumptions for cancer treatment decisions in older populations. Attitudes are ascribed a functional role in guiding behaviour, particularly when confronted with decision-making under conditions of uncertainty (5). Reaching agreement on treatment decisions under these conditions may, therefore, be more complex when attitudes differ between HCPs and patients and could directly impact treatment and subsequent health outcomes (6, 7).

Attitudes are reflected in an individual's preferences, inclinations and tendencies (5). When patient and HCP attitudes and consequent goals differ, this can impact patient representation in treatment decision making (8). The significance of this issue may be heightened in cancer cases, where decisions frequently involve multidisciplinary teams (MDTs) without direct patient involvement. For these cases, HCPs serve as proxies for the patient, emphasising the importance of aligning the understanding of both HCP and patient preferences. If there are discrepancies in priorities between the two, health management decisions may fail to adequately reflect the holistic needs of the patient (9). This, in turn, could ultimately impact the cancer outcomes of the patient as they may not be as invested or adhere to treatment that does not represent their needs or preferences (10).

HCPs often lack sufficient training that fosters a deliberate exploration of older adults' preferences during the decision-making phase of treatment (11). This may lead to the tendency of assuming patient values based on the HCP's personal attitude – informed by their preferences, experiences, and values - which may not accurately reflect patient perspectives (5, 12).

While most studies and recent reviews have focused on older adults' preferences or patient-HCP comparisons (6,9,10,12,13), there is limited research on oncology-based HCP preferences alone.

As our understanding of the older adults with cancer's preferences develops, it is important to clarify the HCP perspective to ensure that the treatment decision process – which is often different for older adults compared to younger adults (14, 15) – is not inhibited by incompatible goals. Consequently, gaining an understanding of the patterns within these attitudes is crucial to identify strategies that can effectively engage healthcare professionals and ultimately benefit older adults with cancer.

For peer review only

Objectives:

This scoping review aims to provide an overview of the attitudes of Healthcare Professionals in treatment decision making related to older adults with cancer.

Specifically:

- Identify patterns in attitudes of HCPs specific to treatment decision making for older adults with cancer
- Identify potential contributing factors to these attitudes
- Highlight gaps in the literature

Methods and analysis:

This protocol is written based on PRISMA ScR guidelines for Scoping reviews (16).

Design

A scoping review will be conducted based on the Arksey and O'Malley methodological framework with the extension developed by Levac and colleagues (17, 18). The reporting of the review will be based on PRISMA ScR guidelines for Scoping reviews (16). A preliminary search - conducted in May 2023 - of PubMed, the Cochrane Database of Systematic Reviews, PROSPERO and Open Science Framework was conducted and no current or underway systematic reviews or scoping reviews on the topic were identified.

Patient and Public Involvement

No patient involved.

Protocol Registration

The final protocol is registered on the Open Science Framework at https://doi.org/10.17605/OSF.IO/T7FD3

Eligibility Criteria

The eligibility criteria was formulated based on the PICO Framework (19), see Table 1.

For peer review only

Table 1: Eligibility criteria based on the PICO framework

Element	Inclusion Criteria	Exclusion Criteria
Participants	Papers involving Healthcare Professionals (e.g. Oncologists, nurses, physicians, nurse practitioners…) of Older Adults (mean/median ≥65 years old) with cancer, any HCP affiliated with cancer care or treatment decisions in cancer care of older adults. The disease focus is on cancer with no restriction on cancer type.	HCPs in the study are exclusively associated with patients younger than 65 years old (for example, paediatric, patient-HCP comparative studies where mean/median patient age <65).<br>HCPs or patients in studies related to diseases other than cancer.
Intervention or exposure of interest	The primary focus of the studies must be on the HCP perspective of treatment decision making in the field of geriatric oncology. Furthermore, the outcomes of the studies should include information regarding subjective inclinations, rankings and perspectives that influence the choices made within the decision-making process. This will be indicative of HCP preferences, attitudes and their individual perspectives.	

For peer review only

**Comparator**	Different HCPs (e.g. Oncologists and specialist nurses). Or different age groups – but this must include a group where the adults are ≥65 years old.<br>Comparisons between patient and HCP priorities, only HCP priorities will be extracted from these studies.<br>Comparisons are not mandatory for inclusion.	None
**Outcome**	Any outcomes related to individual professional factors within treatment decision making in geriatric oncology such as attitudes, perceptions, priorities, or preferences.<br><br>For the purposes of this review, 'attitudes' refers to any subjective inclinations, rankings, and perspectives that influence the choices made when determining the most appropriate course of treatment for their older patients. This description may be encompassed by terms such as perceptions, priorities, or preferences.	
Study Design	Any quantitative design<br>Any qualitative design<br>Mixed methods designs<br>All types of reviews (i.e. literature, narrative, systematic…) to synthesise commonly identified inclinations of HCPs during the treatment decision making process.	Case reports

For peer review only

Article Type	Original peer-reviewed research articles.	Perspective or opinion pieces, book chapters, dissertations, editorials, protocols, conference abstracts
Language	No restriction*	
Study Period	No restriction	
Geography	No restriction	

*Authors' institution hosts diverse language expertise to support this criteria

For peer review only

Information Resources

We will search for papers from the following databases:

- PubMed
- Elsevier Embase
- Medline (from Embase)
- EBSCO CINAHL Complete

Search

The search strategy was developed by a health focused research librarian. Comprehensive search strategies will be employed in each of the databases, utilising a combination of interface command language and text key-words. The search will be formulated in PubMed, from which the strategy will be translated into other databases reflecting their command languages if this is applicable. The search strategy will be made using Boolean operators and truncation. All searches will be reported within PRISMA-ScR guidelines (16).

Table 2: Key concepts and preliminary search terms

**Key concept**	**Search Terms**	**MeSH Terms**

Healthcare professionals	“healthcare professional*” OR “health care professional*” OR “health* professional*” OR “health profession*”	Health Personnel
Treatment decision making	“decision making”, “treatment decision”, “decision making” AND treatment	Decision Making, Clinical Decision-Making
Older adults	Aged, “Older adult*”, Elderly, Geriatric, Nonagenarian, Octogenarian, Centenarian	Aged
Cancer	Cancer*, neoplasm*, tumour*, tumor*, Neoplasia*, Malignanc*, *carcinom*, sarcom*	Neoplasms

Due to the limited number of hits from the above search, no search terms for the key concept of ‘attitudes’ were included.

Study Selection

The selection of studies will follow a multi-stage process:

- *Screening by Title and Abstract*: Prior to primary screening, duplicate records will be removed. Titles will be screened by independent review done in a reviewer pair. Disagreements regarding the inclusion of papers for full text review will be resolved by a third reviewer.
- *Screening by Full text:* Full texts of all papers included after the title and abstract screening will be acquired and assessed by the reviewer pair independently, judging whether the papers meet the inclusion criteria. Disagreements will be resolved through the same process as the title and abstract screening.
- *Screening by References:* The reference list of all included full papers will be screened by the reviewers.

Screening will be streamlined by using a specific review software, Rayyan (20).

For peer review only

Data Management (included and excluded papers)

All papers considered for full text screening during the abstract and title screening process that are then excluded during the full text screening will be tabulated on a digital spreadsheet with a justification for exclusion based upon the inclusion and exclusion criteria. Endnote20 will be used for reference management and bibliography generation.

Abstract-Only Cases

In cases where only the abstract of a potentially eligible paper is available, the authors of this paper will be contacted inquiring whether a full version of the manuscript has been published elsewhere. If it has not been published elsewhere, the study will be excluded. The time limit placed on this correspondence will be 2 weeks. Following this, one follow up email will be sent with the same correspondence time limit. If there is no response, the paper will be excluded.

Data collection process and extraction

Software will be used to compile the reviewer team's extracted data. For every included paper, two reviewers will independently extract the following data as a minimum:

- Author(s)
- Year of publication
- Study objective
- Study design (e.g. qualitative, mixed methods, longitudinal etc.)
- Country of study
- Sampling method (e.g. convenience, snowballing etc.)
- Sample size
- Sample description (sex/gender, age, health profession etc.)
- Description of attitudes if provided
- Descriptions of factors accounted for in the decision making process if provided
- Analytical approach used (e.g. statistical modelling, thematic analysis etc.)
- Summary of main findings

For disagreement over any data point, the same review process followed during screening will be employed.

All collected data will be tabulated for presentation in the written report, accompanied by a narrative summary relating the results to provide an overview of the attitudes of HCP in treatment decision making related to older adults with cancer.

Assessment of Quality

No quality assessment for included papers will be conducted, as suggested by both the Arksey and O'Malley Framework and Joanna Briggs Institute (17, 21).

For peer review only

Implications:

Findings of this review will provide insight into areas of focus to improve the treatment decision making process for older adults with cancer, including variables that may be valuable in the design of future interventions for HCPs involved in geriatric oncology.

Ethics and Dissemination:

No ethical approval is required for this research as it involves the analysis of secondary data. The results of this review will be published in a peer-reviewed journal and presented at conferences.

References:

1. Pilleron S, Sarfati D, Janssen-Heijnen M, Vignat J, Ferlay J, Bray F, et al. Global cancer incidence in older adults, 2012 and 2035: A population-based study. International Journal of Cancer. 2019;144(1):49-58.

2. Berben L, Floris G, Wildiers H, Hatse S. Cancer and Aging: Two Tightly Interconnected Biological Processes. Cancers. 2021;13(6):1400.

3. Boakye D, Rillmann B, Walter V, Jansen L, Hoffmeister M, Brenner H. Impact of comorbidity and frailty on prognosis in colorectal cancer patients: A systematic review and meta-analysis. Cancer Treatment Reviews. 2018;64:30-9.

4. Outlaw D, Williams GR. Is the lack of evidence in older adults with cancer compromising safety? Expert Opinion on Drug Safety. 2020;19(9):1059-61.

5. Riemer H, Shavitt S, Koo M, Markus HR. Preferences don't have to be personal: Expanding attitude theorizing with a cross-cultural perspective. Psychological Review. 2014;121:619-48.

6. Montgomery AA, Fahey T. How do patients' treatment preferences compare with those of clinicians? Qual Health Care. 2001;10 Suppl 1(Suppl 1):i39-43.

7. Neal D, Morgan JL, Kenny R, Ormerod T, Reed MW. Is there evidence of age bias in breast cancer health care professionals' treatment of older patients?. European Journal of Surgical Oncology. 2022 Dec 1;48(12):2401-7.

8. Lamb B, Mostafid H, Vincent C, Green J. Quality improvement in multidisciplinary cancer teams: an investigation of teamwork and clinical decision-making and cross-validation of assessments. Annals of surgical oncology. 2011;18:3535-43.

9. Sathanapally H, Sidhu M, Fahami R, Gillies C, Kadam U, Davies MJ, et al. Priorities of patients with multimorbidity and of clinicians regarding treatment and health outcomes: a systematic mixed studies review. BMJ Open. 2020;10(2):e033445.

For peer review only

10. Smaje A, Weston-Clark M, Raj R, Orlu M, Davis D, Rawle M. Factors associated with medication adherence in older patients: A systematic review. AGING MEDICINE. 2018;1(3):254-66.

11. Back AL, Fromme EK, Meier DE. Training clinicians with communication skills needed to match medical treatments to patient values. Journal of the American Geriatrics Society. 2019 May;67(S2):S435-41.

12. Seghers P, Wiersma A, Festen S, Stegmann ME, Soubeyran P, Rostoft S, et al. Patient Preferences for Treatment Outcomes in Oncology with a Focus on the Older Patient-A Systematic Review. Cancers (Basel). 2022;14(5).

13. Rocque GB, Rasool A, Williams BR, Wallace AS, Niranjan SJ, Halilova KI, et al. What Is Important When Making Treatment Decisions in Metastatic Breast Cancer? A Qualitative Analysis of Decision-Making in Patients and Oncologists. The Oncologist. 2019;24(10):1313-21.

14. Step MM, Siminoff LA, Rose JH. Differences in Oncologist Communication Across Age Groups and Contributions to Adjuvant Decision Outcomes. Journal of the American Geriatrics Society. 2009;57(s2):s279-s82.

15. Sifer-Rivière L, Girre V, Gisselbrecht M, Saint-Jean O. Physicians' perceptions of cancer care for elderly patients: a qualitative sociological study based on a pilot geriatric oncology program. Crit Rev Oncol Hematol. 2010;75(1):58-69.

16. . Tricco AC, Lillie E, Zarin W, O'Brien KK, Colquhoun H, Levac D, et al. PRISMA Extension for Scoping Reviews (PRISMA-ScR): Checklist and Explanation. Ann Intern Med. 2018;169(7):467-73.

17. Arksey H, O'Malley L. Scoping studies: towards a methodological framework. International Journal of Social Research Methodology. 2005;8(1):19-32.

18. Levac D, Colquhoun H, O'Brien KK. Scoping studies: advancing the methodology. Implementation Science. 2010;5(1):69.

19. Schardt C, Adams MB, Owens T, Keitz S, Fontelo P. Utilization of the PICO framework to improve searching PubMed for clinical questions. BMC Med Inform Decis Mak. 2007;7:16.

20. Ouzzani M, Hammady H, Fedorowicz Z, Elmagarmid A. Rayyan—a web and mobile app for systematic reviews. Systematic reviews. 2016 Dec;5:1-0.

21. Peters MD, Godfrey CM, Khalil H, McInerney P, Parker D, Soares CB. Guidance for conducting systematic scoping reviews. Int J Evid Based Healthc. 2015;13(3):141-6.

Authors' contributions:

For peer review only

The scoping review protocol was written up by India Pinker and reviewed by Sophie Pilleron.

Funding

This work is supported by the Luxembourg National Research Fund (FNR), Project n°16731054. The funder did not play a role in the write up of this protocol.

Competing Interests

There are no competing interests to declare.

For peer review only

PRISMA-P 2015 Checklist

This checklist has been adapted for use with protocol submissions to *Systematic Reviews* from Table 3 in Moher D et al: Preferred reporting items for systematic review and meta-analysis protocols (PRISMA-P) 2015 statement. *Systematic Reviews* 2015 **4**:1

Section/topic	#	Checklist item	Information reported		Line number(s)
			Yes	No	
**ADMINISTRATIVE INFORMATION**					
**Title**					
Identification	1a	Identify the report as a protocol of a systematic review	☒	☐	2
Update	1b	If the protocol is for an update of a previous systematic review, identify as such	☐	☒	Not appropriate
**Registration**	2	If registered, provide the name of the registry (e.g., PROSPERO) and registration number in the Abstract	☒	☐	49-50
**Authors**					
Contact	3a	Provide name, institutional affiliation, and e-mail address of all protocol authors; provide physical mailing address of corresponding author	☒	☐	5, 10-11, 15
Contributions	3b	Describe contributions of protocol authors and identify the guarantor of the review	☒	☐	255
**Amendments**	4	If the protocol represents an amendment of a previously completed or published protocol, identify as such and list changes; otherwise, state plan for documenting important protocol amendments	☐	☒	Not appropriate
**Support**					
Sources	5a	Indicate sources of financial or other support for the review	☒	☐	257
Sponsor	5b	Provide name for the review funder and/or sponsor	☒	☐	257
Role of sponsor/funder	5c	Describe roles of funder(s), sponsor(s), and/or institution(s), if any, in developing the protocol	☒	☐	258
**INTRODUCTION**					
**Rationale**	6	Describe the rationale for the review in the context of what is already known	☒	☐	61-94
**Objectives**	7	Provide an explicit statement of the question(s) the review will address with reference to participants, interventions, comparators, and outcomes (PICO)	☒	☐	97-103
**METHODS**					

For peer review only

BioMed Central The Open Access Publisher

For peer review only - http://bmjopen.bmj.com/site/about/guidelines.xhtml

Section/topic	#	Checklist item	Information reported: Yes	Information reported: No	Line number(s)
**Eligibility criteria**	8	Specify the study characteristics (e.g., PICO, study design, setting, time frame) and report characteristics (e.g., years considered, language, publication status) to be used as criteria for eligibility for the review	☒	☐	118 - 126
**Information sources**	9	Describe all intended information sources (e.g., electronic databases, contact with study authors, trial registers, or other grey literature sources) with planned dates of coverage	☒	☐	127 - 132
**Search strategy**	10	Present draft of search strategy to be used for at least one electronic database, including planned limits, such that it could be repeated	☒	☐	134 - 144
STUDY RECORDS					
Data management	11a	Describe the mechanism(s) that will be used to manage records and data throughout the review	☒	☐	158 -162
Selection process	11b	State the process that will be used for selecting studies (e.g., two independent reviewers) through each phase of the review (i.e., screening, eligibility, and inclusion in meta-analysis)	☒	☐	145 - 158
Data collection process	11c	Describe planned method of extracting data from reports (e.g., piloting forms, done independently, in duplicate), any processes for obtaining and confirming data from investigators	☒	☐	169 – 171 & 186 - 190
**Data items**	12	List and define all variables for which data will be sought (e.g., PICO items, funding sources), any pre-planned data assumptions and simplifications	☒	☐	172 - 185
**Outcomes and prioritization**	13	List and define all outcomes for which data will be sought, including prioritization of main and additional outcomes, with rationale	☒	☐	172 - 185
**Risk of bias in individual studies**	14	Describe anticipated methods for assessing risk of bias of individual studies, including whether this will be done at the outcome or study level, or both; state how this information will be used in data synthesis	☒	☐	191 - 193
DATA					
**Synthesis**	15a	Describe criteria under which study data will be quantitatively synthesized	☐	☒	Not appropriate
	15b	If data are appropriate for quantitative synthesis, describe planned summary measures, methods of handling data, and methods of combining data from studies, including any planned exploration of consistency (e.g., I^2, Kendall's tau)	☐	☒	Not appropriate
	15c	Describe any proposed additional analyses (e.g., sensitivity or subgroup analyses, meta-regression)	☐	☒	Not appropriate
	15d	If quantitative synthesis is not appropriate, describe the type of summary planned	☒	☐	188 -190
**Meta-bias(es)**	16	Specify any planned assessment of meta-bias(es) (e.g., publication bias across studies, selective reporting within studies)	☒	☐	191 - 193
**Confidence in cumulative evidence**	17	Describe how the strength of the body of evidence will be assessed (e.g., GRADE)	☒	☐	191 - 193

For peer review only

BioMed Central
The Open Access Publisher
